# Biomarkers of Browning in Cold Exposed Siberian Adults

**DOI:** 10.3390/nu12082162

**Published:** 2020-07-22

**Authors:** Agrafena Efremova, Georgia Colleluori, Mikhail Thomsky, Jessica Perugini, Marina Protasoni, Marcella Reguzzoni, Andrea Faragalli, Flavia Carle, Antonio Giordano, Saverio Cinti

**Affiliations:** 1 Yakut Scientific Center of Complex Medical Problems, 677010 Yakutsk, Russia; a.efremova01@mail.ru (A.E.); ogus@mail.ru (M.T.); 2 Center for the Study of Obesity, Department of Experimental and Clinical Medicine, Marche Polytechnic University, Via Tronto 10A, 60020 Ancona, Italy; g.colleluori@pm.univpm.it (G.C.); j.perugini@univpm.it (J.P.); a.giordano@univpm.it (A.G.); 3 Department of Medicine and Surgery, University of Insubria, Via Ravasi 2, 21100 Varese, Italy; marina.protasoni@uninsubria.it (M.P.); marcella.reguzzoni@uninsubria.it (M.R.); 4 Center of Epidemiology, Biostatistics and Medical Information Technology, Marche Polytechnic University, Via Tronto 10A, 60020 Ancona, Italy; a.faragalli@univpm.it (A.F.); f.carle@univpm.it (F.C.)

**Keywords:** thermogenesis, browning, brown adipose tissue, peripheral blood mononuclear cells, cold-exposure, biomarkers

## Abstract

Cold-exposure promotes energy expenditure by inducing brown adipose tissue (BAT) thermogenesis, which over time, is also sustained by browning, the appearance, or increase, of brown-like cells into white fat depots. Identification of circulating markers reflecting BAT activity and browning is crucial to study this phenomenon and its triggers, also holding possible implications for the therapy of obesity and metabolic diseases. Using RT-qPCR, we evaluated the peripheral blood mononuclear cells (PBMC) expression profile of regulators of BAT activity (*CIDEA*, *PRDM16*), white adipocytes browning (*HOXC9* and *SLC27A1*), and fatty acid β-oxidation (*CPT1A*) in 150 Siberian healthy miners living at extremely cold temperatures compared to 29 healthy subjects living in thermoneutral conditions. Anthropometric parameters, glucose, and lipid profiles were also assessed. The cold-exposed group showed significantly lower weight, BMI, hip circumference, and PBMC expression of *CIDEA*, but higher expression of *HOXC9* and higher circulating glucose compared to controls. Within the cold-exposed group, BMI, total cholesterol, and the atherogenic coefficient were lower in individuals exposed to low temperatures for a longer time. In conclusion, human PBMC expresses the brown adipocytes marker *CIDEA* and the browning marker *HOXC9*, which, varying according to cold-exposure, possibly reflect changes in BAT activation and white fat browning.

## 1. Introduction

Obesity is a multifactorial chronic disease whose prevalence is ~20% and ~40% in Europe [1] and United States [2], respectively. During the last decades, the worldwide increase in obesity incidence made the need for effective therapeutic strategies extremely urgent, especially in consideration of its comorbidities and associated mortality [1,2,3,4]. Obesity is characterized by an aberrantly elevated amount of white adipose tissue (WAT) resulting from a dysfunctional regulation of the energy balance [5]. The modulation of energy intake and expenditure is extremely complex and results from the integration of numerous neuroendocrine and environmental signals [6,7]. Cold-exposure is one of the environmental stimuli promoting energy expenditure through the activation of thermogenic pathways, a crucial response to ensure survival to hostile temperature conditions [6,7]. Specifically, cold promotes β-adrenergic stimulation via the sympathetic nervous system (SNS), which in turn induces thermogenesis by activating brown adipose tissue (BAT) [6,7,8]. BAT burns fat to produce heat given the expression of UCP1 [6]. Interestingly, upon cold stimulation, white adipocytes can transdifferentiate into beige (also known as “brite”) adipocytes (brown-like phenotype with elevated expression of UCP1) in a process known as “browning,” leading to heat production [9,10]. Importantly, during the phenomenon of browning, proliferation and differentiation of brown adipocyte precursors also occurs, contributing to the expansion of the heat-producing cell population [5,11]. In mice, BAT activation was shown to protect against obesity, type 2 diabetes and atherosclerosis [12,13]. Hence, the study of BAT regulation has been particularly attractive as a potential target for obesity treatment [5,14]. Human adults have varying amounts of BAT which decreases with advancing age and BMI [15,16]. The study of BAT activation and browning in humans is not simple due to several limitations. The most used procedure available for this purpose is the study of (18)F-FDG (2-deoxy-2-[^18^F]fluoro-d-glucose) uptake by Positron-Emission Tomography-Computed Tomography (PET-CT) which, besides different technical limitations, is costly and complex [15,17,18]. Alternative techniques to assess BAT activation and WAT browning are needed. Studies by Palou and colleagues conducted on female rats demonstrated that the expression of regulators of BAT activity (*Cidea*, *Prdm16*), WAT browning (*Hoxc9* and *Slc27a1*), and fatty acid β-oxidation (*Cpt1a*) in both tissues, correlates with the expression of the same modulators in the peripheral blood mononuclear cells (PBMC) upon cold stimulation [19]. The authors concluded that these genes could be considered suitable brown/beige markers to be assessed in PBMC, avoiding the use of invasive procedures [19]. However, it is unknown whether brown/beige markers’ expression in human PBMC is detectable and whether it varies depending on cold-exposure. We recently showed that cold-exposed Siberian adults (living outdoor) display greater β-adrenergic activation and browning of visceral adipose depots compared to individuals living in thermoneutral conditions [20]. The main objective of our study was to evaluate whether PBMC of cold-exposed Siberian miners expresses different levels of the brown/beige and fatty acid utilization markers compared to the PBMC of Siberian adults living in thermoneutral conditions. Our secondary aim was to evaluate differences in the metabolic status between our groups under investigation.

## 2. Materials and methods

### 2.1. Study Design

This was an observational, cross-sectional study. The study was conducted in 2013 in the Verkhoyansky and Anabrasky Districts of Yakutia, following the guidelines in the Declaration of Helsinki for the ethical treatment of human subjects. The protocol was approved by the local committee review board (Supplemental File S1). 

### 2.2. Study Participants

In this study, we recruited 150 healthy male diamond miners engaged in open-pit diamond mining at Anabrasky District of Yakutia (Polar Zone, cold-exposed group) and healthy control subjects living in the town-center of Verkhoyansky, District of Yakutia (urban area) in thermoneutral conditions. Subjects enrolled in the cold-exposed group spent an average time of 8 h per day working in the mine for 3 months (December to February) when the average temperature is −45/−52 °C [21]. Cold-exposed individuals lived close to the mining camp in dedicated accommodations in the countryside. At the time of recruitment, the study coordinator conducted a questionnaire-based interview to estimate the amount of time spent in cold conditions based on professional responsibilities. For the cold-exposed group, all blood sampling and anthropometric evaluations were performed in the camp or miners’ dwellings. Twenty-nine healthy male subjects from the same region were enrolled in the control group during summer (months of August) when the average temperature is +16 °C to +18 °C. Similarly, at the time of recruitment, the study coordinator conducted a questionnaire-based interview to make sure that none of the individuals belonging to the control group was exposed to cold during the last three months before enrollment. For this purpose, a questionnaire formulated ad hoc by the research team was used. Control subjects were living in the urban area, where the impact of cold during the winter months is minimal due to the presence of heating systems in houses and in means of transportation (+20–25 °C). Based on the data collected during the interview, winter months cold exposure for the control group was estimated to be 20–30 min per day maximum and was mainly due to brief walks in open areas. The study coordinator contacted the manager of the miners’ company “Anabar Diamonds” and got permission to access the mining camp for cold-exposed subjects’ recruitment. Healthy control subjects were recruited through the walk-in clinic of the Scientific Center for Complex Medical Problem of Yakut. Subjects were eligible to be enrolled in the control group if they did not suffer from any chronic disease and if they were not taking any medication or undergoing any kind of medical treatment. Medical information of study subjects belonging to the cold-exposed group was provided by the medical doctor of the working camp, whose role was to take care of workers’ health. Individuals with a documented diagnosis of any chronic or metabolic disease such as metabolic syndrome, type 2 diabetes, dyslipidemia or taking any medication that could affect glucose or lipid metabolism were excluded from this study. Before enrollment in the study, all participants signed a written consent form. 

### 2.3. Anthropometric Evaluation

At the time of recruitment, body weight and height were measured by the standard weighing scale and stadiometer, respectively. BMI (kg/m^2^) was calculated by dividing the weight (in kilograms) by height (in meters) squared. Waist circumference (cm) was measured in a standing position midway from the lower edge of the costal arch to the iliac crest of the ilium bone. Hip circumference (cm) was measured in the standing position at the level of the greater trochanters of the femurs.

### 2.4. Blood Sampling and Biochemical Evaluations

Blood samples were collected in the morning between 8:00 and 11:00 AM from study subjects who were asked to fast overnight. Samples collection was performed during February for the cold-exposed group and in August for the control group, after 3 months of exposure to cold and thermoneutral conditions, respectively. After collection, samples were immediately frozen at −60 °C and transported to the laboratory of the Yakut Scientific Center of Complex Medical Problems, Yakutsk Russian Federation. Samples collection for the control group was performed at the clinical laboratories of the Scientific Center for Complex Medical Problem of Yakut. Control glucose, triglycerides, total cholesterol and high-density lipoprotein (HDL) cholesterol measurements were determined using an automated biochemical analyzer Labio 200 (Mindray Medical International Limited, Nanshan, Shenzhen 518057, China) using Biocon kits (Biocon, Electronic City, 560100 Bangalore India). Low-density lipoprotein (LDL) and very-low-density lipoprotein (VLDL) cholesterols were assessed using the following formulas: LDL = total cholesterol-VLDL-HDL; VLDL = (TG)/2,2. The atherogenic coefficient ka was calculated using the formula Ka = (total cholesterol-HDL)/HDL [22]. The optimal coefficient of atherogenity is considered to be between the value 2 and 3; where values higher than 3, predispose individuals to elevated risk for atherosclerosis and cardiovascular events.

### 2.5. PBCM Collection and qPCR

Whole blood samples were collected into EDTA coated vacutainers and immediately transported to the laboratory for further processing. PBMCs were isolated by gradient separation using OptiPrep™ medium (D1556, Sigma-Aldrich, St. Louis, MO, USA), according to manufacturer’s instructions, with modifications previously described by Paolu and colleagues [19]. Briefly, blood was filled up to 6 mL with solution C (146 mM NaCl and 1 mM HEPES). Blood was then layered to form a density barrier by mixing 2.7 mL of OptiPrep medium with 9.3 mL of OptiPrep diluent without intermixing (3 mL of density barrier per 2 mL of blood-solution C mixture) in a centrifuge tube. Afterward, the tube was centrifuged at 700× *g* for 20 min at 20 °C with acceleration and deceleration adjusted at zero. The layer containing PBMCs and platelets was collected from the interface between plasma layer and OptiPrep medium. To wash PBMCs and to remove the platelets, the collected material was centrifuged in solution C at 400× *g* for 10 min at 20 °C. Samples were kept at −70 °C before RNA isolation. Total RNA extraction was performed using the RNeasy Plus Minikit (Qiagen, Hilden, Germany, Cat.#74134) following the manufacturer instructions. In brief, 8 mL of human peripheral blood were added with RBC Lysis Buffer to a final volume of 45 mL and incubated at room temperature for 10 min. Cells were pelleted by centrifugation at 600× *g* for 10 min. The supernatant was removed, and the pellet was resuspended in 1 mL of RBC Lysis Buffer. Afterward, cells were pelleted and subsequently resuspended in 1 mL DPBS and pelleted again by centrifugation. The pellet was resuspended in 1200 μL of TRIzol and 0.2 mL of Chloroform and vortexed for 15 s. Samples were centrifuged at 13,000 rpm for 10 min at 4 °C. The upper phase was transferred to clean microcentrifuge tubes and an equal volume of cold isopropanol was added to the mixture. The tubes were inverted several times and placed in a −20 °C freezer for precipitation. Samples were centrifuged at 13,000 rpm for 10 min at 4 °C. The supernatant was then carefully removed, and the pellet was rinsed with 0.5 mL of ice-cold ethanol (75%). Samples were then centrifuged at 13,000 rpm for 10 min at 4 °C, the supernatant was removed, and samples were let dry at room temperature for 10 min. The RNA pellet was dissolved in 20 μL of RNAse-free water.

RNA quality was assessed with an IMPLEN P-300 nanophotometer. After quantitation, the RNA samples were stored at −80 °C before proceeding with retrotranscription. Fifty nanograms of total RNA from PBMCs were reverse transcribed into cDNA using the iScript cDNA synthesis kit (Bio-Rad Laboratories, Hercules, CA, USA, Cat.#1708891) following the manufacturer’s instructions. The reaction was performed using a T-100 Thermal Cycler (Bio-Rad), and the conditions were: 25 °C for 5 min, 42 °C for 30 min, and 85 °C for 5 min. Gene expression was assessed by RT-qPCR using the SFX96 Real-Time System. Target genes were chosen based on the study by Palou et al., who identified markers detectable in rats PBMC [19]. Gene functions and selected primers are described in Table 1. Each PCR reaction mix included diluted (1:5) cDNA template, forward and reverse primers (1μM), SYBR Green PCR Master Mix (Bio-Rad, Cat.#1725272) and nuclease-free water to a total volume of 20 μL. PCR reaction conditions were as follows: 15 s at 95 °C, 1 min at 60 °C, and 15 s at 95 °C. Gene expression data for each target are expressed as relative quantification (ΔΔCT) adjusted for the housekeeping gene GAPDH (forward primer GTCGGAGTCAACGGATTTGGT; reverse primer AGTGATGGCA TGGACTGT).

### 2.6. Statistical Analysis

The normality of the variables was assessed through the Kolmogorov–Smirnov test. A non-parametric approach was followed due to the small sample size. Median and interquartile ranges (IQR) were used to summarize the variables. The Wilcoxon sum-rank test was used to evaluate differences between the two groups. In order to investigate differences between groups, the non-parametric ANCOVA with smoothed regression and Young and Bowman test was applied. Markers and biochemical variables were the dependent variables, and age and BMI were the covariates. One model for each dependent variable was performed. The non-parametric ANCOVA was also applied to evaluate the effect of the number of cold-exposure hours on the distribution of each marker and biochemical variables in cold exposed subjects, using BMI as covariate. Based on the cold-exposure time distribution, four classes were considered: 1 or 2 h, 4 or 4.5 h, 8 or 10, 11 h. Benjamini–Hochberg *p*-value adjustment method was applied. For the biochemical variables, the interaction between classes of cold exposure and BMI was also considered.

## 3. Results

A total of 179 subjects were enrolled in this study: 29 of them belonged to the control group, while 150 were miners belonging to the cold-exposed group. The distributions of each variable were asymmetric; hence, a non-parametric statistical approach was chosen. All subjects were male with median age equal to 32 years (IQR: 28; 38). Among the cold-exposed individuals, 35% were exposed for less than 5 h, with 21% of subjects exposed for less than 2 h; 65% were exposed to cold for more than 5 h, with 55% exposed for 11 h. Table 2 shows the characteristics of the enrolled subjects. In brief, no significant differences were found for age, waist circumference (WC), and waist to hip circumferences ratio (W/H) between the two groups. Meanwhile, weight, height, BMI, and hip circumference were significantly lower in the cold-exposed group compared to the controls (Table 2).

The PBMC gene expression study revealed significantly lower *CIDEA* and higher *HOXC9* expression levels in the cold-exposed group compared to the control group, while no significant differences in other markers’ expression were detected (Table 3). After adjustment for age (and with and without adjustment for BMI), circulating total cholesterol, LDL, HDL, VLDL, and triglycerides, as well as the atherogenic coefficient were comparable between the two groups in analyses. However, the cold-exposed group had significantly higher circulating glucose levels compared to the control group (Table 3).

Table 4 shows the result of the analysis conducted on the cold exposed group, and due to the number of markers’ missing values, we compared the subjects exposed to cold for less than 11 h and the subjects exposed to cold for 11 h. No clear trend in the adjusted medians and no statistically significant differences in the distribution of markers were observed. This could be attributed to the high variability of markers distribution in each cold-exposure group. A statistically significant difference among cold-exposure groups was observed for BMI, total cholesterol, and the atherogenic coefficient, for which the adjusted medians decreased when the number of cold-exposure hours increased.

## 4. Discussion

This is the first study investigating the expression of browning, beige, and fatty acids utilization regulators in the PBMC of human subjects chronically exposed to extremely cold temperatures compared to controls living in thermoneutral conditions. Cold-exposed subjects expressed lower levels of the brown adipocytes’ marker *CIDEA* and higher levels of the beige adipocytes’ marker *HOXC9* compared to controls, while the expression of the other investigated genes did not differ significantly between groups. Interestingly, cold-exposed individuals from this study had higher circulating glucose, but lower body weight, BMI and hip circumference compared to controls, possibly reflecting a healthier metabolic status. Our data proved that certain brown and beige adipocytes markers are detectable in human PBMC and vary according to cold exposure, potentially reflecting changes in BAT activation, WAT browning, and related metabolic status.

The increased energy expenditure associated with cold-induced BAT activation and WAT browning has attracted enormous interests for its potentials in the treatment of obesity and metabolic diseases [5,16,23,24]. During the last decades, in fact, numerous efforts have been made to identify browning regulators and alternative stimuli responsible for BAT activation [5,14,16,23,25,26,27]. However, since only invasive techniques are available for the assessment of browning [17], the study of this phenomenon in humans presents important limitations. Hence, the identification of circulating markers of browning or of browning-induced metabolic changes would be extremely useful. Several circulating “batokines” have been discovered [28], although none of them has been recognized as a valid marker of BAT activation. Interestingly, Paolu et al. demonstrated that cold-induced changes in the expression of few genes regulating browning, beiging, and fatty acid oxidation in rats’ BAT and WAT are reflected by changes in the expression of the same regulators in the PBMC, pointing the attention to new potential analytical candidates [19]. Nevertheless, this finding has never been explored and validated in humans.

We recently demonstrated that cold-exposed Siberian adults (living outdoor) display higher browning of visceral adipose depots compared to individuals living in thermoneutral conditions [20]. The aim of this study was to explore whether Siberian subjects belonging to the same population also exhibit a differential PBMC expression of the markers identified by Palou and colleagues. Cold-exposed subjects enrolled in our study expressed higher levels of the beiging’s marker *HOXC9* and lower amounts of the brown adipocytes’ marker *CIDEA* compared to controls. This finding is in some ways consistent with the ones of Palou and colleagues whose study was conducted on female rats of different ages (1, 2, 4, and 6 months) exposed to cold for one week [19]. Consistently to our results, in fact, cold exposure lead to a significant increase in the PBMC *Hoxc9* expression of adult rats (4 and 6 months) [19]. This marker is considered to be specific to the beige adipose depots, and its expression is known to increase upon browning stimulation (rosiglitazone administration) [29]. The increase of *HOXC9* in the PBMC of our cold-exposed human subjects could thereby reflect the expression changes in their adipose depots, making it a potential circulating candidate to be used as a browning marker. Our data are consistent with the findings from RNA sequencing studies showing that human BAT has a gene expression signature resembling the one of beige adipocytes [30]. On the other hand, Palou and colleagues did not detect significant changes in adult rats PBMC expression of *Cidea* exposed to cold, but they only revealed an evident reduction in the mRNA levels of the marker in the BAT [19]. *Cidea* is widely expressed on the surface of lipid droplets of brown adipocytes and is responsible for the formation of large lipid droplets through the promotion of lipid exchange between them [31,32]. Adipocytes’ *Cidea* expression increases in conditions that favor triglycerides deposition [32], an opposite phenomenon compared to what happens during BAT activation. According to some studies, in fact, it antagonizes UCP1 expression [33]. *CIDEA* lower expression in the PBMC of our cold-exposed group compared to controls could reflect similar differences in this marker’s BAT expression, whose levels reduced following exposure to low temperatures in animal models [19,34]. The evaluation of PBMC expression of *CIDEA* has the potential of great clinical relevance in the study of human BAT activation that needs further exploration. In our study, we could not detect differences in the PBMC expression of *CPT1A4, SCL27,* and *PRDM16* comparing cold exposed individuals to controls. This finding is in contrast with the study of Palou and colleagues who observed an increased expression in *Cpt1a4* and *Scl27* in female adult rats PBMC upon cold exposure [19]. The differences in our findings and the one of Palou could be attributed to several elements, for example, the diverse experimental models, gender, age, conditions under investigation, and the variability of these markers in the studied groups.

The cold-exposed group of the present study also had lower body weight, BMI, and hip circumference, possibly reflecting a healthier metabolic status [35]. Although we did not detect significant differences in cholesterol levels between our two groups, our data revealed lower levels of BMI, total cholesterol, and atherogenic coefficient with increasing daily time of exposure to cold. This finding is consistent with evidence reporting increased lipid utilization and improved lipid profile induced by BAT activation produced by cold stimulation [13,36,37,38]. On the other side, other reports revealed a U-shaped relationship between environmental temperature and cardiovascular risk, with the last one increasing for temperatures lower than −1 °C and higher than 20 °C [39]. However, most of these studies analyzed temperatures ranging from ~−15 to 30 °C, different from our study, in which cold exposed individuals were exposed to temperatures lower than −30 °C. Furthermore, our cold-exposed group had higher fasting glucose levels compared to controls. Although this finding may seem counterintuitive, considering that acute BAT activation increases glucose uptake [37,38], plasma glucose levels do not change upon acute cold-exposure in humans [37,38]. Thus, it is possible that in individuals with normal glycemia, BAT chronic stimulation requires higher basal glucose levels for its usage, without leading to metabolic abnormalities (impaired fasting glucose or insulin resistance). Importantly, the fasting glucose levels of both of our groups were within the range of normality.

Our study has several limitations. Although we studied a unique population exposed to extremely cold temperature, the cross-sectional nature of our investigation does not allow us to establish a cause-effect relationship between the variables under analysis. Furthermore, since we investigated Siberian, male adults adapted to live in very cold temperatures, we do not know if our findings can be extended to different ethnicities, gender, or ages. Our data need to be validated by additional long-term studies with a larger sample size, evaluating in parallel BAT and PBMC gene expression and BAT activation through multiple techniques. Furthermore, data regarding the metabolic status of this population, such as insulin sensitivity and the existing correlation with the metabolic profile, should be investigated more in-depth.

## 5. Conclusions

In conclusion, this is the first study demonstrating that human PBMC expresses markers of brown adipocytes and browning and that *CIDEA* and *HOXC9* mRNA levels vary according to cold-exposure. Based on our results, we believe that *CIDEA* expression in human PBMC could mirror its expression in BAT in a condition of chronic activation, while *HOXC9* expression could mirror the one of white adipocytes undergoing white-to brown transdifferentiation, making both markers potentially useful circulating indexes of BAT and browning activation deserving further investigation and validation.

## Figures and Tables

**Table 1:** Brown, beige, and fatty acid utilization markers under investigation.

Gene	Function	Primers
*CIDEA*	Encoding for a protein recognized as a marker of brown adipose tissue	Forward:ATCGGCTCCTTAACGTGAA Reverse:AACCGCAGCAGACTCCTCA
*PRDM16*	Encoding for a protein regulating brown adipose tissue differentiation	Forward:CCCAACAAGTACAGCCTGGA Reverse:GCGGATGAGGTTGGACTTCC
*SLC27A1*	Encoding for a protein recognized as a marker of beige/brite adipocytes (white in brown) conversion	Forward:GCGATATACCAGGAGCTGCA Reverse:TCTTGAAGGTGCCTGTGGTG
*HOXC9*	Encoding for a protein marker of beige/brite adipocytes (white in brown) conversion	Forward:CAGCAACCCCGTGGCC Reverse:CCGAGGTCCCTGGTTAAA
*CPT1A4*	Encoding for a protein (liver isoform) involved in the mitochondrial oxidation of long-chain fatty acids	Forward:TCCACGA TTCCACTCTGCTC Reverse:CAGCAACCCCGTGGCC

**Table 2:** Characteristics of subjects according to cold exposure.

	Control *n* = 29	Cold-Exposed *n* = 150	*p*
Age [years, median (IQR)]	34 (29; 38)	32 (28; 38.5)	0.522
Height [cm, median (IQR)]	174 (171; 176)	172 (168; 176)	0.011
Weight [kg, median (IQR)]	75 (73; 81)	70 (64; 78)	<0.001
BMI [kg/m^2^, median (IQR)]	25.61 (23.84; 27.04)	24.06 (22.15; 26.51)	0.023
WC [cm, median (IQR)]	92 (84; 96)	85 (78; 95)	0.082
HC [cm, median (IQR)]	100 (99; 102)	95 (92; 101)	0.001
WC/HC [median (IQR)]	0.91 (0.85; 0.93)	0.89 (0.85; 0.95)	0.872

*p*-value refers to the Wilcoxon test; IQR: 1st–3rd quartiles. BMI: body mass index; HC: hip circumference; WC: waist circumference.

**Table 3:** PBMC gene expression of markers of browning, beigeing, and fatty acid utilization and biochemical variables, comparison according to cold exposure.

	Control *n* = 29	Cold-Exposed *n* = 150			
	Adj. Median (CI 95%)	Adj. Median (CI 95%)	RSE	Coefficient	*p* *
**PBMC Markers**					
*CIDEA*	0.49 (0.43; 0.58)	0.30 (0.22; 0.43)	0.467	0.019	0.042
*PRDM16*	2.97 (1.99; 3.64)	1.74 (0.66; 3.42)	3.727	0.362	0.622
*SLC27A1*	1.30 (0.92; 1.88)	1.12 (0.73; 1.78)	2.772	0.125	0.761
*HOXC9*	0.96 (0.61; 1.18)	1.75 (0.90; 2.42)	2.891	0.271	0.038
*CPT1A4*	2.40 (1.73; 2.83)	2.30 (0.55; 2.99)	4.167	0.155	0.931
**Biochemical Variables**					
Glucose (mmol/L)	4.42 (4.35; 4.69)	5.29 (5.22; 5.36)	0.851	0.121	0.025
Triglycerides (mmol/L)	1.27 (1.00; 1.61)	1.22 (1.08; 1.47)	0.563	0.016	0.763
Total cholesterol (mmol/L)	5.12 (4.71; 5.54)	4.99 (4.81; 5.17)	0.728	0.019	0.888
LDL (mmol/L)	3.30 (2.95; 3.63)	3.08 (2.79; 3.20)	0.842	0.191	0.856
HDL (mmol/L)	1.21 (1.02; 1.41)	1.59 (1.13; 1.99)	0.534	0.011	0.557
VLDL (mmol/L)	0.71 (0.58; 0.84)	0.55 (0.49; 0.62)	0.474	0.021	0.899
Ka	3.42 (2.56; 4.51)	3.33 (2.61; 4.07)	0.062	0.062	0.878

RSE refers to Residual Standard Error; Adj. Median refers to the adjusted median of smoothed regression; CI 95% refers to a 95% Confidence Interval. * non-parametric ANCOVA test via smoothing regression with Benjamini–Hochberg’s *p*-value adjustment; each model was adjusted for age and BMI. Ka: atherogenic coefficient.

**Table 4:** PBMC markers and biochemical variables comparison within the cold exposed group based on time of exposure to cold.

	1–2 h		4–4.5 h		8–10 h		11 h		*p*
	*n* = 31		*n* = 21		*n* = 14		*n* = 82		
BMI [Median (CI 95%)]	31	26.26 (23.51; 28.54)	20	25.51 (24.57; 26.39)	14	24.22 (23.70; 25.39)	82	23.12 (21.73; 24.49)	<0.05
**Markers**									
*CIDEA* [Adj. median (CI 95%)]	1	-	8	0.36 (0.11; 0.82)	7	0.33 (0.01; 0.848)	22	0.30 (0.02; 0.58)	0.974 ^†^
*PRDM16* [Adj. median (CI 95%)]	15	0.64 (0.12; 2.55)	16	2.57 (0.74; 4.39)	8	0.85 (0.15; 3.46)	43	1.77 (0.64; 2.89)	0.684 ^†^
*SLC27A1* [Adj. median (CI 95%)]	6	0.18 (0.01; 2.74)	7	1.68 (0.05; 4.14)	8	3.05 (0.78; 5.26)	20	1.20 (0.03; 2.59)	0.799 ^†^
*HOXC9* [Adj. median (CI 95%)]	24	2.02 (0.61; 3.42)	14	1.78 (0.11; 3.50)	11	3.33 (1.24; 5.41)	52	1.68 (0.72; 2.64)	0.524 ^†^
*CPT1A4* [Adj. median (CI 95%)]	9	0.27 (0.08; 3.44)	9	2.58 (0.15; 5.80)	8	4.25 (0.86; 7.64)	22	2.29 (0.26; 4.32)	0.668 ^†^
**Biochemical variables**									
Glucose (mmol/L) [Adj. median (CI 95%)]	31	5.10 (4.74; 5.47)	20	5.28 (4.86; 5.70)	14	5.46 (4.91; 6.02)	82	5.20 (4.98; 5.42)	0.831
Triglycerides (mmol/ L) [Adj. median (CI 95%)]	31	0.96 (0.75; 1.18)	20	0.98 (0.74; 1.23)	14	1.29 (0.96; 1.61)	82	1.22 (1.09; 1.35)	0.217
Tot cholesterol (mmol\L) [Adj. median (CI 95%)]	31	4.99 (4.71; 5.27)	20	4.63 (4.31; 4.96)	14	4.39 (3.96; 4.72)	82	4.43 (4.16; 4.68)	<0.05
LDL (mmol/L) [Adj. median (CI 95%)]	31	2.93 (2.58; 3.27)	20	3.22 (2.83; 3.62)	14	2.87 (2.36; 3.89)	82	3.10 (2.90; 3.31)	0.881
HDL (mmol/L) [Adj. median (CI 95%)]	31	1.78 (1.35; 2.16)	20	1.19 (0.95; 1.49)	14	1.26 (0.93; 1.68)	82	1.38 (1.25; 1.51)	0.679
VLDL (mmol/L) [Adj. median (CI 95%)]	31	0.45 (0.35; 0.55)	20	0.48 (0.36; 0.59)	14	0.56 (0.41; 0.72)	82	0.56 (0.50; 0.62)	0.558
Ka [Adj. median (CI 95%)]	31	3.34 (2.97; 3.72)	21	3.04 (2.62; 3.46)	14	2.61 (2.04; 3.18)	82	2.64 (2.41; 2.86)	<0.05

CI 95% refers to 95% Confidence Interval; *p* refers to non-parametric ANCOVA test via smoothing regression with Benjamini–Hochberg’s *p*-value adjustment; each model was adjusted for and BMI. Ka: atherogenic coefficient. No adjusted median and CI 95% were reported because only 1 subject was in the subgroup. ^†^ Comparison between group of subjects exposed to cold for less than 11 h and subject exposed to cold for 11 h.

## Supplementary Materials

The following are available online at https://www.mdpi.com/2072-6643/12/8/2162/s1, File S1: Protocol.
