# Supplementary material for: Biomarkers of Browning in Cold Exposed Siberian Adults
Source: Nutrients. 2020 Jul 22;12(8):2162. doi: 10.3390/nu12082162 (PMC7468804; doi:10.3390/nu12082162)
Supplement: Supplementary file 1 [file nutrients-12-02162-s001.pdf]

Yakut scientific center
of complex medical problems
Sergeliakhskoe road 4th km., Yakutsk, 677010
Fax: (411-2)32-19-81
E-mail: ysckmp@yandex.ru
TIN 1435122648
Code of reason for registration: 13501001

Local committee of bio-medical ethic
Statement from protocol No. 46 from May 24, 2018
Decision No. 7

Present: Zakharov Y.S., Sidorova O.G., Kononova S.I., Fedorova S.I., Soloveva N.A., Kononova S.K.
Considered: protocol of scientific research No. 209 "**Gene expression in brown fat tissue and in blood of people of Yakutia in natural cold conditions".**
Performer: Efremova Agrafena Vladimirovna
Scientific center: Yakut scientific center of complex medical problems, University of Ancona (Politecnica delle Marche) (Ancona, Italy)
Following documents were presented to ethic committee for consideration:

1. Application for expertise of research protocol;
2. Research protocol No. 209;
3. Professional auto biography of researcher;
4. Form of informational consent of research;

Resolved:
The aim of research is exposure of oblique (Cidea, Cpt1a, Hoxc9, Prdm16, Slc29a1) and straight gene – sharpie (UCP-1) presence of active brown fat tissue of people of Yakutia in natural cold conditions. Participation to research process with maintenance of ethic norms under informational consent. Members of ethic committee give approval for research project.

Head of Ethic committee	<signed>	S.K. Kononova
Secretary	<signed>	M.N. Mikhailova

[stamp]: Federal Agency of scientific organization
Federal State Budgetary scientific institution
Yakut scientific center of complex medical problems

The translation is done by Aiza Y. Kytakhova, a professional translator of the Centre of Foreign Languages, Yakutsk, Russia from Russian into English. Diploma 101404 0005625, registration № 1119, issued by

Federal State Autonomous Educational Institution of Higher Education “North-Eastern Federal University named after M. K. Ammosov” Yakutsk. The translation corresponds to the original text in full.
March 28, 2019
Настоящий перевод выполнен профессиональным переводчиком «Центра изучения иностранных языков», Кытаховой Айзой Юрьевной, с русского на английский. Диплом Федерального государственного автономного образовательного учреждения высшего профессионального образования «Северо-Восточный федеральный университет имени М.К. Аммосова» 101404 0005625, регистрационный № 1119. Перевод в полном объеме соответствует тексту оригинала.
28 Марта, 2019 г.

Подпись переводчика/Translator ___________________________ (Кытахова Айза Юрьевна)
